# Anesthesia for patients with *PTRF* mutations: a case report

**DOI:** 10.1186/s40981-017-0139-9

**Published:** 2018-01-25

**Authors:** Atsuko Hirano, Tomohiko Takada, Mariko Senda, Hidemasa Takahashi, Takeo Suzuki

**Affiliations:** Department of Anesthesiology, Tokyo Metropolitan Bokutoh Hospital, 4-23-15 Kotobashi, Sumida-ku, Tokyo, 130-8575 Japan

**Keywords:** PTRF mutation, General anesthesia, Malignant hyperthermia, Long QT syndrome, Lipodystrophy, Neuromuscular disease

## Abstract

**Background:**

Polymeraze I and transcript release factor (*PTRF*) mutations are a newly recognized disease, which cause congenital generalized lipodystrophy associated with myopathy.

**Case presentation:**

A 29-year-old man (height 126 cm; weight 22 kg) with a *PTRF* mutation was scheduled for mandibular dentigerous cystectomy. His primary symptoms were lipodystrophy, myopathy, long QT syndrome, refractory nephrosis, and abnormal lipid metabolism. Defibrillator pads were applied soon after the patient entered the operating room. Anesthesia was induced using continuous administration of dexmedetomidine (4 μg/kg/h) for 15 min; midazolam (7 mg) was added while monitoring the bispectral index and his vital signs. Remifentanil and rocuronium were administered before endotracheal intubation. The surgeon used local anesthesia, and dexmedetomidine and remifentanil were titrated throughout the surgery. The surgery was performed uneventfully, and the patient was extubated following the administration of sugammadex and flumazenil.

**Conclusion:**

Patients with *PTRF* mutations require careful anesthetic planning. We planned to administer lipid-free, non-inhalational agents for the induction and maintenance of anesthesia. The anesthetic method used for this minor surgery was safe and effective.

## Background

Caveolae are 50- to 100-nm invaginations of cell-surface membranes [1], characterized by the presence of the membrane protein caveolin. Caveolae are involved in many cellular processes, including cell signaling, lipid regulation, and endocytosis [2]. Among the caveolin isoforms, caveoline-1 (Cav1) and caveoline-2 (Cav2) are expressed in most cell types. Cav1 is involved in cholesterol transport and signal transduction, whereas caveoline-3 (Cav3) is specific to skeletal, smooth, and cardiac muscle and results in muscular disorders when defective [3].

The polymerase I and transcript release factor (PTRF) protein plays an essential role in the formation of caveolae as well as in the stabilization of caveolins [4]. Recently, a new disease caused by *PTRF* mutations has been reported, mainly characterized by congenital generalized lipodystrophy with onset during infancy. Other potential complications include skeletal dysplasia, abnormal metabolism of lipid and glucose, skeletal muscle abnormalities (such as muscular hypertrophy, atrophy, or mounding), cardiac conditions (such as cardiomyopathy and long QT syndrome [LQTS]), immunodeficiency, growth disturbance, and renal impairment [4–6]. Providing general anesthesia to patients with *PTRF* mutations is challenging, given their high risk of developing malignant hyperthermia (MH), their abnormal lipid metabolism, and their risk of developing life-threatening arrhythmias related to LQTS.

Few reports on patients with *PTRF* mutations have been published to date [4–6], and no guidelines for providing anesthesia in such patients have yet been reported. Here, we report our clinical experience with successfully providing general anesthesia to a patient bearing a *PTRF* mutation.

## Case presentation

A 29-year-old man with a *PTRF* mutation (height 126 cm; weight 22 kg) was scheduled for mandibular dentigerous cystectomy. He was born with neonatal asphyxia and was diagnosed with non-Fukuyama muscular dystrophy at 4 months of age. At 3 years of age, he began treatment for nephrosis. By 19 years of age, he had experienced two syncopal episodes. Holter electrocardiography led to a diagnosis of LQTS, for which the patient was started on antiarrhythmic drugs. At 22 years of age, he was diagnosed using genetic analysis with a caveolin-related metabolic disorder caused by a *PTRF* mutation. By 29 years of age, he developed cholecystitis and visited our hospital. The cholecystitis was not clinically urgent, but a purulent dentigerous cyst extending to the bone was found that required treatment. The attending physician in charge of dental oral surgery requested assistance from the Department of Anesthesiology.

**Fig. 1:**
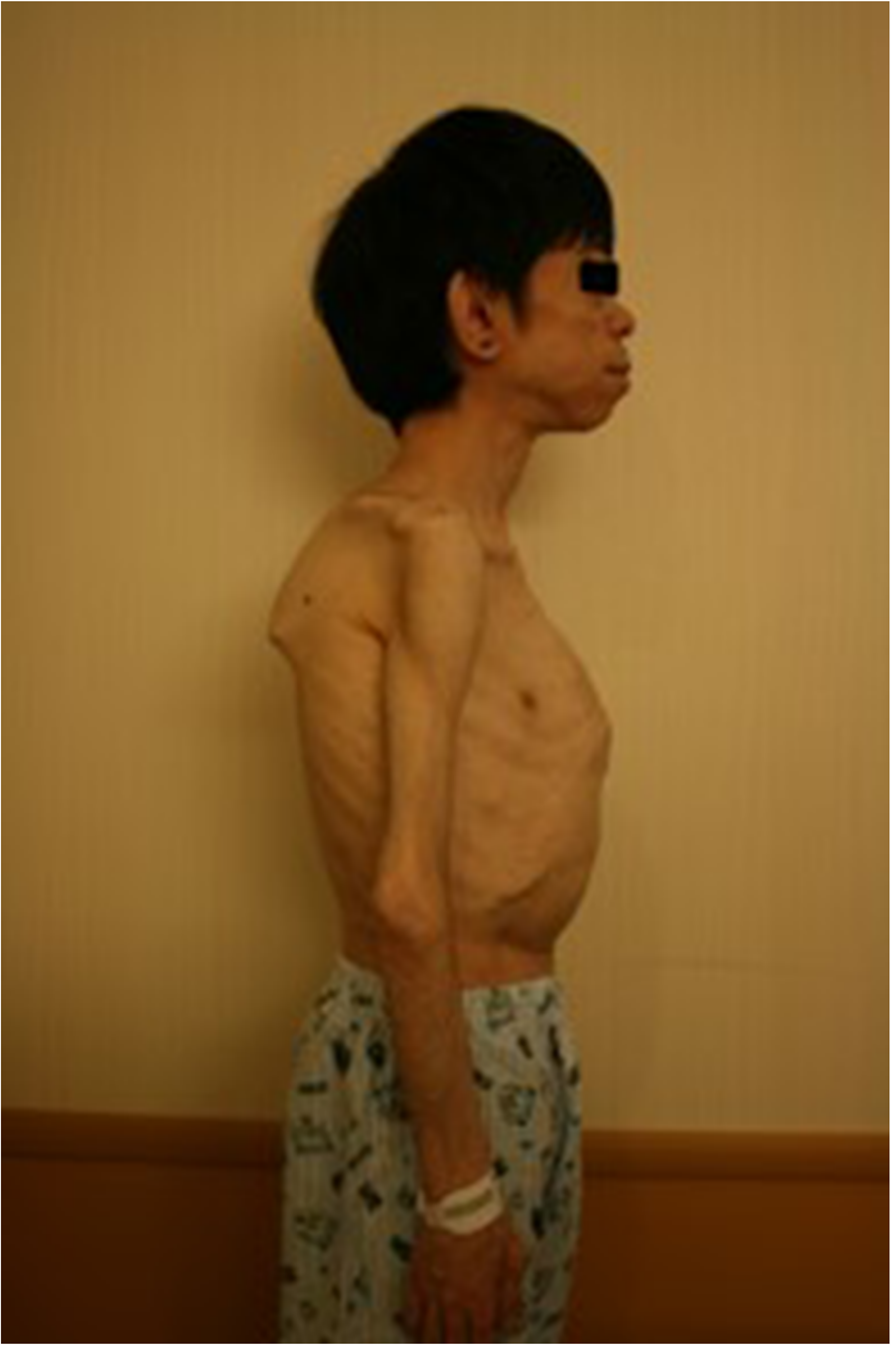
His appearance from the side

**Fig. 2:**
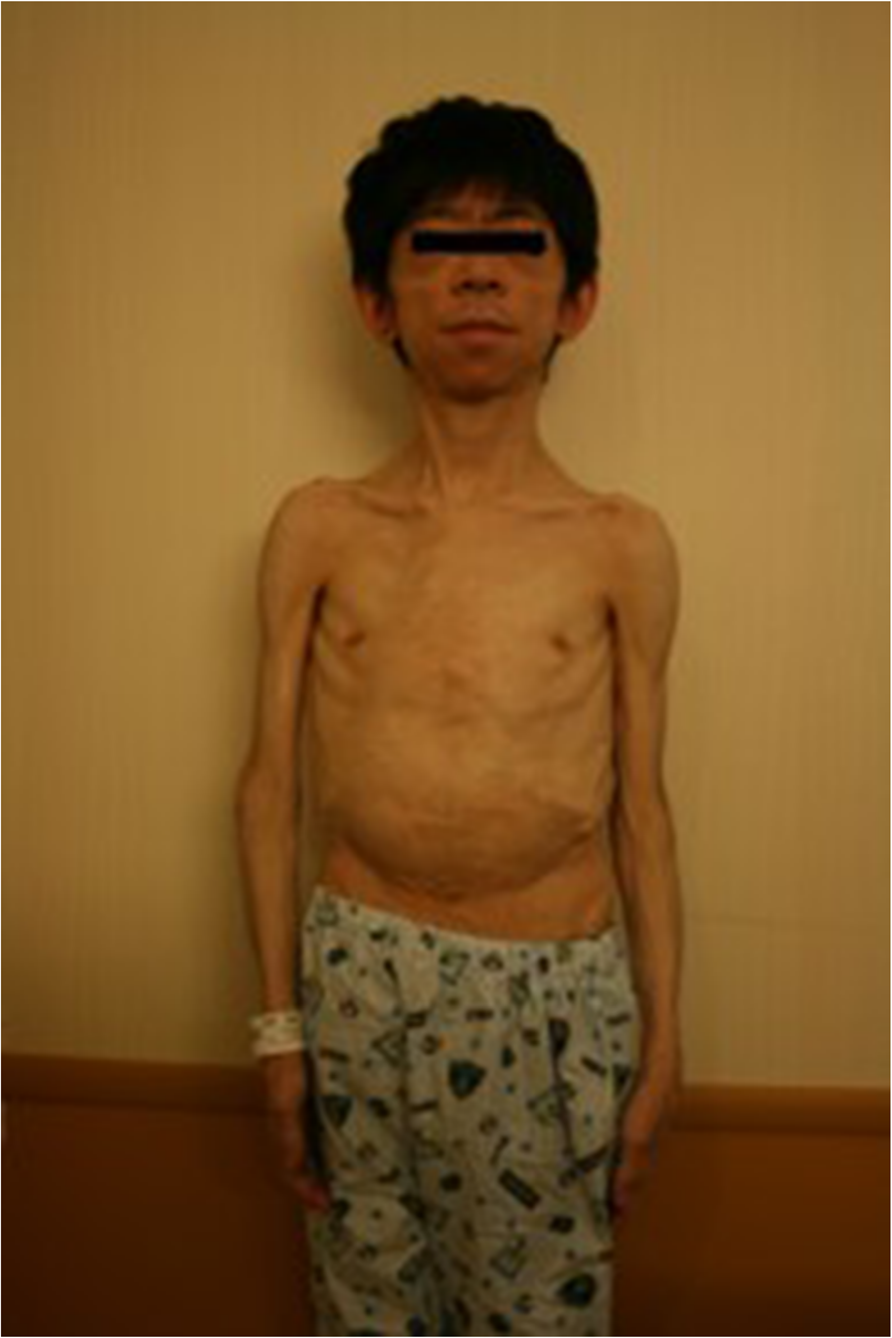
His appearance from the front

The patient’s primary symptoms were congenital generalized lipodystrophy associated with myopathy, LQTS, refractory nephrosis, abnormal lipid metabolism, and skeletal dysplasia (Figs. 1 and 2). He was taking methylprednisolone 4 mg/day, mexiletine 300 mg/day, propranolol 30 mg/day, levocarnitine 500 mg/day, alfacalcidol 1 μg/day, cyclosporine A 10 mg/day, and pravastatin 5 mg/day. We observed a generalized lack of subcutaneous fat on physical examination. Further, the patient reported that he was performing all his day-to-day activities without any assistance, and that he had no dietary restrictions.

Serum investigations revealed elevated levels of creatine kinase (282 U/L), total cholesterol (243 mg/dL), and triglycerides (272 mg/dL), as well as normal glucose level (91 mg/dL). Renal dysfunction was well controlled by medication, and his estimated glomerular filtration rate was within normal limits (275.5 mL/min/1.73 m^2^). A preoperative resting electrocardiograph showed a sinus rhythm, heart rate of 66 bpm, and a QT/QTc ratio of 370/383, which were normal. Cardiac function was also normal according to transthoracic echocardiography. Using a chest radiograph, we measured his narrowest tracheal diameter (14.5 mm) to select the size of the endotracheal tube. His pulmonary function tests showed a vital capacity of 1.92 L (69.8% of predicted) and a forced expiratory volume of 1.90 L (67.6% of predicted).

In planning anesthesia for this patient, we first considered avoiding volatile agents to prevent MH. Moreover, there were concerns about drug selection, given his abnormal lipid metabolism. We also required a plan in the event of a life-threatening arrhythmia due to LQTS. Furthermore, skeletal dysplasia may cause difficulty in securing the airway, and the use of a muscle relaxant was a concern due to his myopathy.

We opted for total intravenous anesthesia (TIVA) without using propofol, which has a high lipid content. We chose dexmedetomidine and midazolam as sedative drugs and remifentanil as an analgesic. A bispectral index (BIS™) monitor (MEDTRONIC, Minneapolis, MN, USA) was used to titrate each agent. We planned not to use muscle relaxant, but to handle a situation where administration of a muscle relaxant would be required, rocuronium was prepared, which could be reversed by sugammadex. We also planned to apply defibrillation pads throughout the 24-h perioperative period and had antiarrhythmic drugs ready for use in the event of a life-threatening arrhythmia. We prepared a McGrath® video laryngoscope for intubation. In case it did not fit, we had also prepared a pediatric Airway Scope® (PENTAX, Tokyo, Japan).

On the day of surgery, we first washed out volatile anesthetics from the anesthesia machine by flushing the system with oxygen (10 L/min for 30 min). We applied defibrillator pads soon after the patient entered the operating room due to his risk of developing a life-threatening arrhythmia. We also administrated atropine 0.01 mg/kg to avoid the occurrence of long QT due to bradycardia. The BIS score was 97 before the induction. Anesthesia was induced with continuous administration of dexmedetomidine 4 μg/kg/h for 15 min followed by midazolam 1−2 mg according to the BIS score and his vital signs. Lidocaine was sprayed around his larynx. The BIS score was 69 after when we administered midazolam up to 7 mg. After confirming that mask ventilation is possible, we administrated remifentanil 2 μg/kg/h and then performed laryngoscopy, but he had a strong gag reflex with 69 of the BIS. We then administered additional remifentanil (2 μg/kg), but the BIS score was still high (64) and he still had a gag reflex. Since we anticipated that administration of a large amount of remifentanil may cause extreme bradycardia, we decided to use a muscle relaxant instead of administering additional remifentanil. We administered an initial dose of rocuronium (5 mg), and an additional dose (5 mg), which helped relieve his gag reflex, and the BIS score decreased to 50. We placed an endotracheal tube (internal diameter 6 mm) using a McGrath® video laryngoscope without difficulty. Throughout the induction, there were no cardiovascular or oxygenation-related complications. For maintenance of anesthesia, the surgeon performed a mandibular nerve block using 1% lidocaine hydrochloride-epinephrine (9 mL), and we continued to titrate dexmedetomidine 0.7 μg/kg/h and remifentanil 0.15–0.25 μg/kg/min according to the invasiveness of the procedure, his BIS score, and his vital signs. Further, in order to avoid bradycardia, we did not administer additional remifentanil; however, this proved difficult because his BIS score remained high (60–80) during the surgery. We monitored his rectal temperature throughout the surgery, it was 35–36.5 °C throughout the surgery.

The surgery was completed in 34 min, with 51 mL of blood loss. Before extubation, we administered sugammadex and complete reversal of neuromuscular blockade was confirmed with a train-of-four (TOF) monitor (TOF-Watch®, MSD K.K. Kenilworth, NJ, USA). After the surgery was finished, his BIS score decreased and remained around 60 for more than 15 min. As the patient took longer than usual to regain consciousness, we used flumazenil to reverse the effects of midazolam. Soon after receiving flumazenil, his responsiveness and BIS score improved to 95, and he was extubated and transferred to the intensive care unit. Since the half-life of flumazenil is shorter than that of midazolam, we continued administering flumazenil for 3 h, reducing the dose by half every hour, to maintain consciousness and a BIS score > 90. Additionally, the defibrillation pads were retained until the next day. Despite using a non-standard anesthetic method and although the BIS score remained high during the surgery, the patient reported feeling comfortable and unaware during surgery at the next day post-operative visit. He was discharged without any complications.

## Discussion

Cav3 is suspected to be one of the causes of MH due to its role in stabilizing the junction between the plasma membrane and sarcoplasmic reticulum by interacting with one of the Ca2+ channels and RYR1 [7]. Since *PTRF* mutations can cause a deficiency in Cav3, these patients are considered to be at risk of developing MH. We therefore decided to avoid the use of volatile anesthetics to prevent MH.

In general, when avoiding volatile anesthetic agents, propofol is chosen for TIVA because of its short-acting, adjustable effect. In this case, we decided not to use propofol because we did not know whether it was safe to use a lipid-containing agent in this patient. As *PTRF* mutations cause a deficiency of Cav1 and caveolae, the ability of adipocytes to store triglycerides is impaired, which leads to an increase in the levels of circulating lipids. The patient reported that he had no dietary restrictions, but in general, in patients with lipodystrophy, the level of triglycerides should be maintained by limiting the dietary fat to 20–30% of the total energy. Based on the patient’s height and weight, we estimated that he would require 800–1000 kcal per day, with an ideal lipid intake of approximately 20–30 g. We calculated that if we use propofol 1–2 mg/kg for induction and propofol 4–10 mg/kg for maintenance for 1 h, then the amount of lipid intake would be approximately 135–337 g, which is 4 to 16 times the stipulated limit. Furthermore, since we used an intravenous mode of administration, the actual lipid intake amount would be higher than that estimated for oral intake. We used dexmedetomidine, which rarely causes respiratory depression, and midazolam, which can be antagonized by flumazenil to facilitate clear awakening. Intraoperative analgesia was obtained by a mandibular nerve block and remifentanil infusion.

The use of muscle relaxants was another concern because of the possibility of enhancement and prolongation of action of these drugs in patients with neuromuscular diseases. Many recent reports indicate that a combination of rocuronium and sugammadex can be safely used for patients with neuromuscular disorders; therefore, we decided to use rocuronium under TOF monitoring and sugammadex for reversal based on these reports [8, 9].

LQTS and life-threatening cardiac arrhythmia are sometimes observed in patients with *PTRF* mutations, although the underlying mechanism remains unclear [10]. *CAV3* mutations are known to cause LQTS [10, 11], while *PTRF* mutations also lead to a deficiency of Cav3 and caveolae where several cardiac ion channels have been reported to be localized. Furthermore, *PTRF* mutations may also cause cardiac dysfunction accompanied by cardiomyocyte hypertrophy and cardiac fibrosis [10]. These findings indicate that *PTRF* mutations may cause LQTS and life-threatening arrhythmias; thus, it is necessary to take precautionary measures in these patients. In this case, the patient was taking an oral regimen of antiarrhythmic drugs. To counteract a life-threatening arrhythmia due to the stress of surgery and pain, defibrillator pads were applied immediately after he entered the operating room and were retained until the next day when he was discharged from the ICU. Additionally, we attempted to alleviate stress by providing verbal explanations, and we had magnesium and other anti-arrhythmic drugs ready for use throughout the procedure.

In patients with *PTRF* mutations, local anesthesia is recommended whenever possible. When patients need to undergo general anesthesia, careful management is needed.

## Conclusions

In conclusion, patients with *PTRF* mutations have many severe manifestations, although much remains unknown about the conditions caused by these mutations. In this case, the anesthetic approach we chose was effective during the short surgery in this patient.

## Abbreviations

BIS: Bispectral index
LQTS: Long QT syndrome
MH: Malignant hyperthermia
PTRF: Polymerase I and transcript release factor
TOF: Train-of-four
